# Modeling Freight Vehicle Type Choice using Machine Learning and Discrete Choice Methods

**DOI:** 10.1177/03611981211044462

**Published:** 2021-09-23

**Authors:** Usman Ahmed, Matthew J. Roorda

**Affiliations:** 1 Department of Civil & Mineral Engineering, University of Toronto, Toronto, Canada

**Keywords:** vehicle type choice, random forest, machine learning, discrete choice model, urban freight transportation

## Abstract

The choice of vehicle type is one of the important logistics decisions made by firms. The complex nature of the choice process is because of the involvement of multiple agents. This study employs a random forest machine learning algorithm to represent these complex interactions with limited information about shipment transportation. The data are from Commercial Travel Surveys with information about outbound shipment transportation. This study models the choice among four road transport vehicle types: pickup/cube van, single-unit truck, tractor trailer, and passenger car. The characteristics of firms and shipments are used as explanatory variables. SHAP-based variable importance is calculated to interpret the importance of each variable, and shows that employment and weight are the most important variables in determining the choice of vehicle type. The random forest model is also compared with the multinomial and mixed logit models. The model prediction results on the validation data are compared. The results show that random forest model outperforms both the multinomial and mixed logit model with an overall increase in accuracy of about 7.8% and 9.6%, respectively.

The choice of freight mode is one of the key logistics decisions made by firms. The choice of freight mode depends on factors such as commodity attributes, time sensitivity, and the geographical context. Competition among freight modes, such as road transport, rail, marine, and air transport is typically modeled at regional, state, and national geographic levels. However, in an urban context, the choice is among road vehicle types, as in an urban area road transport is usually the only mode available (*1*). Freight vehicles cause emissions, noise, and safety impacts, and are a major reason for pavement damage. Parking is another import issue in urban areas. Freight delivery vehicles face problems in finding parking and often resort to illegal parking spaces. In dense urban areas (such as city centers) some vehicle types are prohibited or have limited access. Therefore, for urban transport, it is important to distinguish freight vehicle types as they have a significant effect on a city’s livability (*1*).

Understanding of the factors that influence vehicle type decisions is important to support infrastructure planning decisions and urban freight policy analysis. These factors include origin and destination location, type of shipment, and industry type. Two main actors are responsible for mode and vehicle type choice decisions: the shipper and carrier firm. Shippers produce the commodity, whereas carriers are responsible for shipment transportation. The choice of mode is determined through an interaction between shipper and carrier firms (*2*). In the case of private fleets, the shipper also plays the carrier role. This study investigates how shipper firms choose vehicle type for shipment transportation in an urban area.

Most studies in the literature focus on freight mode choice and few investigate the choice of freight vehicle type. We provide a literature review of both freight mode choice and vehicle type choice to provide an overview of work done in the two domains. Freight mode choice has been modeled at different geographical levels, using different methods. A review of freight mode choice models is provided by de Jong (*1*). Discrete choice models are the most common method to model freight mode choice. Rich et al. developed a weighted logit mode choice model for the Oresund region, Denmark (*3*). The choice of freight mode between truck, rail, and ship is modeled at the upper level in a nested logit modeling framework, whereas the choice of crossings (ferry route or bridge) is at the lower level. The representative consumers are described by origin–destination (OD) pairs and commodity groups. Arunotayanun and Polak developed multinomial logit (MNL), mixed MNL, and latent class models using stated preference (SP) data collected in Java, Indonesia. The shippers selected freight mode between small truck, large truck, and rail in eight SP choice scenarios (*4*). Samimi et al. developed binary logit and probit models for the choice among rail and truck modes using data from an online national survey conducted in the USA (*5*). Similarly, Wang et al. used binary logit and probit models to assess the choice between truck and rail mode using Freight Analysis Framework (FAF) data for trips originating from the three FAF zones for Maryland, USA (*6*). Keya et al. developed a national level freight mode choice model using a hybrid utility–regret-based approach for the USA (*7*). The study used 2012 Commodity Flow Survey data to model the choice between for-hire truck, private truck, air, parcel or courier service, and other modes. The choice of freight mode is also modeled jointly with shipment size as the two choices could be made jointly. For instance, Pourabdollahi et al. used copula-based joint MNL–MNL model for the choice of freight mode and shipment size (*8*). The study modeled freight mode choice between truck, rail, air, and courier and five classes of shipment size using an establishment-based survey in the US (*8*). Stinson et al. used a nested logit model approach to model the two decisions jointly (*9*). Rail, truck, and parcel/air modes are considered, and shipment size is categorized as small, medium, large, or very large. The study used the 2012 Commodity Flow Survey data for the State of Arizona in the US. Keya et al. used copula-based random regret minimization approach to model freight mode choice and shipment size (*10*). The choice of mode between for-hire truck, private truck, air, parcel or courier service, and other modes is modeled using a MNL model, and the shipment size is modeled using the ordered logit modeling method. The study used the 2012 Commodity Flow Survey of the US.

The studies that modeled freight vehicle type choice include Cavalcante and Roorda (*11*), who developed a discrete/continuous model for the choice of vehicle type and shipment size. The vehicle types considered are passenger car, pickup/van, single-unit truck, and tractor trailers. Nuzzolo and Comi investigated the use of car, light goods vehicle, and medium goods vehicle by retailers using a MNL model (*12*). Irannezhad et al. developed a MNL model of vehicle type as part of a joint copula-based vehicle type and shipment size model (*13*). The vehicle types considered are van, truck, heavy truck, and trailer. Ahmed and Roorda developed MNL and mixed MNL models for vehicle type choice for the Greater Toronto and Hamilton Area (*14*).

Machine learning algorithms have rarely been used to model freight mode choice. To the authors’ knowledge, no study is found in the literature that uses machine learning algorithms to model freight vehicle type choice. The studies that model freight mode choice using machine learning algorithms include Abdelwahab and Sayed which used artificial neural networks (ANNs) (*15*), Tortum et al. which used ANNs and adaptive neuro-fuzzy inference system (ANFIS) (*16*), Syed and Razavi which used ANN and B-spline network-based neurofuzzy approach (*17*), and Syed et al. which used B-spline associative memory network-based fuzzy inference system and ANFIS for the choice between rail and truck mode (*18*).

Machine learning algorithms provide an alternative approach to represent complex relationships within a high dimensional dataset (i.e., many explanatory variables) without any prior assumptions about the data such as distribution of error terms. The calculation of variable importance offers a method to interpret the relative contribution of each explanatory variable, and addresses much of the criticism of machine learning algorithms as black box methods. Machine learning algorithms have been frequently used for mode choice in passenger demand modeling. These machine learning algorithms include ANNs (*19* [Bibr bibr20-03611981211044462]–*21*), decision trees (*22*, *23*), and Bayesian networks (*24*). However, the use of machine learning algorithms to model freight vehicle type choice is still missing.

This study develops a vehicle type choice model using a random forest (RF) machine learning algorithm. Several studies in the transportation-related domain show that RF has better prediction accuracy than other machine learning algorithms. These include travel mode choice (*25* [Bibr bibr26-03611981211044462]–*27*), safety (*28*, *29*), travel time/traffic flow prediction (*30*, *31*), and pattern recognition (*32*, *33*). Therefore, the RF method is selected for this study. The underlying hyperparameter tuning process, which is overlooked in most studies, is also discussed in detail. To assess the performance of the RF model, this study compares the RF model with a MNL model and a mixed MNL that accounts for panel structure of the data. This study focuses on small-to-medium-size firms (employment ranging from 1 to 250) in the Toronto Area, using the data from Commercial Travel Surveys. As the information about vehicle ownership for shipment transportation is not available, we assume that the choice of vehicle type is made by shipper firm even if the vehicle is not owned by the shipper firm. This study has applications in agent-based microsimulation models such as the framework presented by Roorda et al. (*34*).

## Data Source

**Figure 1.**
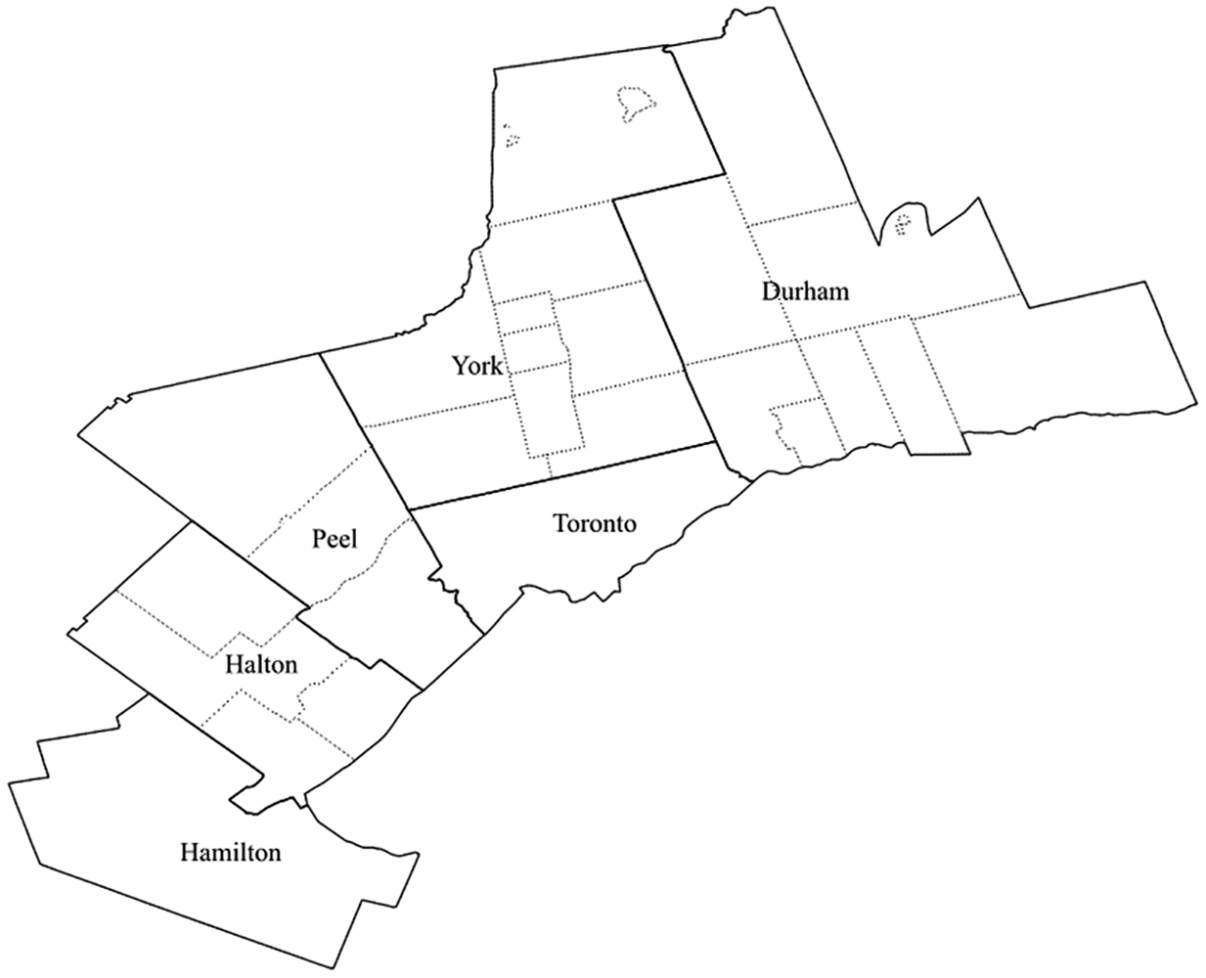
Toronto area.

This study combines data from three Commercial Travel Surveys conducted in the Region of Peel (2006/07), Region of Durham (2010), and the Toronto Area (2012). Figure 1 shows the Toronto Area which contains the City of Toronto and other regions. The solid lines in Figure 1 show regions, whereas the dotted lines within regions show cities/local municipalities. For example, Peel Region contains three cities/local municipalities as shown in Figure 1. The data include firm characteristics, such as employment (ranging from 1 to 250 employees) and industry classification, and outbound shipment characteristics such as commodity type, weight, origin, and destination. Multiple shipments are sent by a firm over the survey day resulting in multiple observations by the same firm. The data consist of 1,439 shipments made by 385 firms. The data over-represent firms in Peel Region. Table 1 shows descriptive statistics of firms and shipments. The firms belong to one of the six industry classifications as shown in Table 1.

**Table 1.** Descriptive Statistics of Explanatory Variables

Variable	Records	Percentage
Firm characteristics		
Industry type		
Wholesale trade and transportation handling (NAICS 41, 48–49)	633	44
Manufacturing (NAICS 31–33)	493	34
Retail trade (NAICS 44–45)	195	14
Construction (NAICS 23)	59	4
Agriculture, forestry, fishing, and hunting (NAICS 11)	34	2
Heavy industry (NAICS 21)	25	2
Shipment characteristics		
Commodity type		
Manufactured products	372	26
Food and food products	276	19
Metal and metal products	225	16
Other	164	11
Non-metallic products	103	7
Road vehicles and parts	92	6
Wood, pulp, and paper	82	6
Chemicals and plastics	39	3
Machinery and electrical	31	2
Textiles	24	2
Mixed	17	1
Agricultural products	14	1
Origin		
Mississauga	772	54
Brampton	146	10
Pickering	86	6
Caledon	70	5
Toronto	56	4
Other	309	21
Destination		
Outside Toronto area	547	38
Toronto	272	19
Mississauga	202	14
Brampton	69	5
Vaughan	45	3
Other	304	24

*Note*: NAICS = North American Industry Classification System

**Figure 2.**
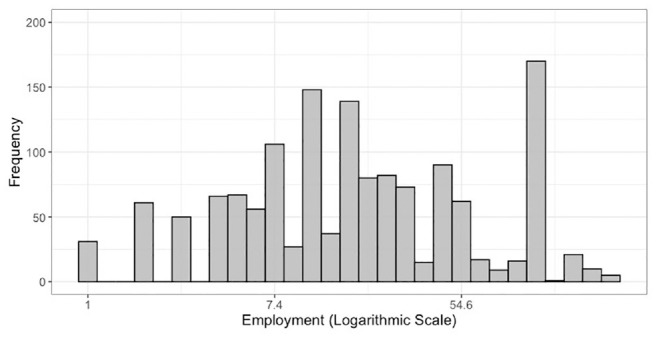
Firm employment distribution.

The corresponding North American Industry Classification System (NAICS 2012) code is also provided based on the description of industries in the survey instrument. Firm employment (number of employees working in the business establishment), shown in Figure 2, ranges from one employee to 250 employees. Larger firms (with employment between 100 and 250 employees) are under-represented, and very large firms with employment greater than 250 are not included in the surveys. We have included firms with employment between 100 and 250, though estimation of models excluding larger firms does not affect the model estimation results. The distribution of employment is skewed; therefore, the natural logarithm of the employment is used in the models.

**Figure 3.**
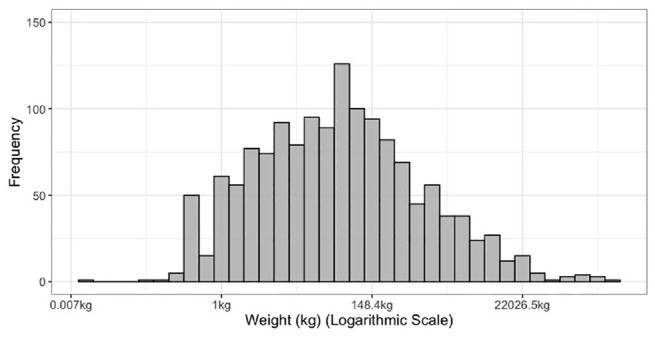
Shipment weight distribution.

Characteristics of the shipment include commodity type, origin, and destination of shipment. A total of 12 commodity types are considered in this study and over half of them are manufactured, food, and metal products as shown in Table 1. The origin and destination of shipments are considered at the level of census subdivision (usually local municipalities/cities) and Table 1 shows the five most frequent origins and destinations out of a total of 21 origins and 26 destinations. Shipments sent outside the study area are characterized as outside Toronto Area. The shipment weight is recorded by the respondent in units such as metric tonnes, imperial tons, pounds, or liters and is converted into kilograms. Similar to employment, the distribution of shipment weight is highly skewed, and therefore the natural logarithmic transformation of shipment weight is used as an explanatory variable. Figure 3 shows the distribution of shipment weight in kilograms. Four vehicle types comprise the dependent variable considered in this study and are listed in Table 2. Pickup or cube van is the most frequent vehicle type, whereas single-unit truck and tractor trailer have similar representation. The vehicle type with the lowest frequency is the passenger car.

**Table 2.** Summary of Vehicle Types Considered in the Study

Vehicle type	Records	Percentage
Pickup or cube van	499	35
Single-unit truck	420	29
Tractor trailer	377	26
Passenger car	143	10

## Methodology

This study compares a RF model with MNL and mixed MNL models. The models are estimated on training data and evaluated on validation data. The data are divided based on firms rather than individual shipments. The training data contain 70% of the firms, that is, 269 firms resulting in 1,114 outbound shipments, and the validation data contain 30% of the firms, that is, 116 firms resulting in 325 outbound shipments. The models are developed based on the best specification of explanatory variables for each model. The best RF model specification is selected based on prediction accuracy and the MNL and mixed MNL model specifications are selected based on t-ratios, overall rho-squared, and Bayesian information criterion (BIC) value. The comparison between the three models is based on the prediction accuracy on the validation data.

### Random Forest

**Figure 4.**
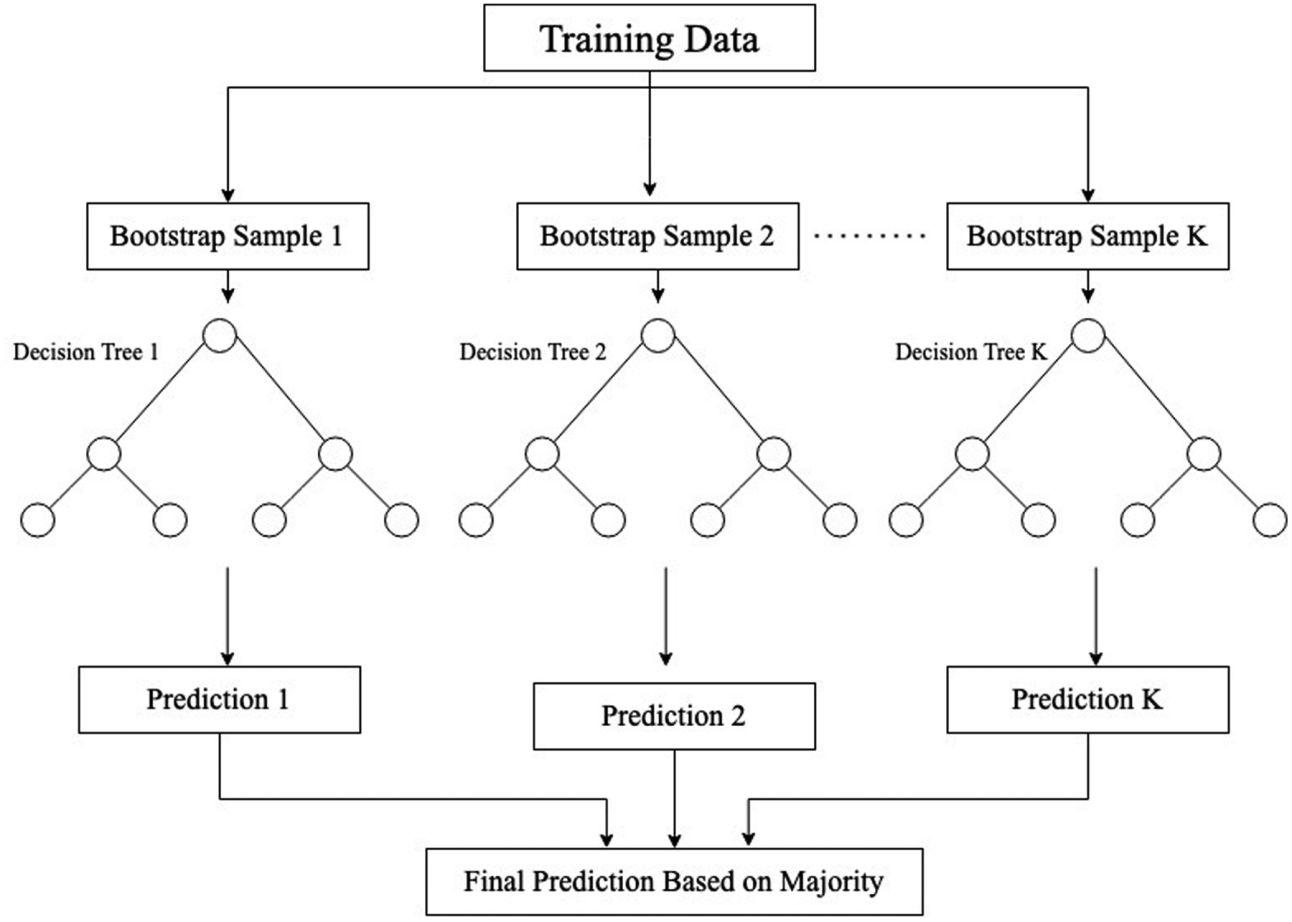
Random forest method illustration.

The RF method is introduced by Breiman (*35*) and consists of a forest of decision trees. It belongs to the bagging class of ensemble learning algorithms. The concept of ensemble-based algorithms is to create several classifiers (such as decision trees) and combine the output to reduce error (*36*). Bagging is a method that creates multiple classifiers by using bootstrap samples (random samples with replacement) and the final output is the one predicted by the majority of the classifiers (*36*). The RF method is illustrated in Figure 4. As different samples are used by each classifier, it creates diversity among classifiers. Additional diversity is attained in the RF method by randomly selecting a group of explanatory variables (sometimes called “predictors” or “features” in the machine learning literature) to find the best split when splitting a node of a decision tree (*37*).

To develop a RF model, three hyperparameters are to be specified. Hyperparameters are parameters that affect the model performance but cannot be learned from the training data. Their values are specified by the modeler. The process of selecting the optimal values of the hyperparameter is called hyperparameter tuning. It involves testing multiple values of the hyperparameters and selecting the optimal values based on a test metric. The three hyperparameters of a RF model are:

- **Trees**: The number of decision trees. An increase in number of trees generally results in a decrease in error but significantly increases the computation time. In this study 1,000 trees are used, as model performance does not improve further beyond 1,000 trees and computational time is reasonable.
- **Mtry**: The number of candidate variables to consider at each node split. This hyperparameter is tuned by testing multiple values.
- **Min_n**: The minimum node size which represents the minimum number of observations in the terminal node of a decision tree. This hyperparameter is tuned by testing multiple values.

The test metric to evaluate the model performance for different hyperparameter values is the model prediction accuracy. A 10-fold cross-validation procedure is used to select the optimal hyperparameter values. The procedure divides the training data into ten parts, trains the data on nine parts and tests the model performance on the remaining tenth part. The procedure is repeated ten times until all the parts are used for training and testing.

### Variable Importance

Model results are interpreted using variable importance. Variable importance is a measure that assesses the impact of an explanatory variable on the model output. The method of SHAP (Shapley additive explanation) (*38*) is used to determine variable importance in this study. SHAP provides the importance of each variable by comparing model predictions with and without the variable using Shapley values (*38*). For SHAP values, the order in which the variables are added could affect their relative contributions. Therefore, all possible combinations are tested, and results are averaged over all possible orderings to provide SHAP value. This process becomes computationally expensive. For tree-based models such as RF, Lundberg et al. recently developed the Tree SHAP method which provides exact tree solutions for SHAP values which reduces the computational time from exponential to polynomial (*39*). SHAP values are calculated for every observation.

SHAP summary plots are used to present the results of all observations. In a SHAP summary plot, variables are sorted by their relative global impact. Each point represents the SHAP value for a variable for a given observation. The color of the point shows its value, with high value represented by red and low value represent by blue color (*39*). For a particular class (i.e., vehicle type), a higher SHAP value shows higher log odds to be classified in that particular class.

### Multinomial Logit Model

The MNL model is based on the utility maximization approach where the utility function for the choice of each vehicle type v by firm *n* for shipment *t* is defined as (*40*):

(1) $$U_{\mathrm{vnt}}=V_{\mathrm{vnt}}+\varepsilon_{\mathrm{vnt}}$$

$V_{\mathrm{vnt}}$ is the systematic component and $\varepsilon_{\mathrm{vnt}}$ is the unobserved component of utility. The unobserved component is assumed to be extreme value (Type I) distributed independently and identically across alternatives v , firms n and shipments *t*. The probability that vehicle type *v* is chosen by firm *n* for shipment *t* becomes (*40*):

(2) $$P_{\mathrm{vnt}}=\frac{e^{V_{\mathrm{vnt}}}}{\sum_{k\in K}e^{V_{\mathrm{knt}}}};\mathrm{where}\;K\;\mathrm{is}\;\mathrm{the}\;\mathrm{set}\;\mathrm{of}\;\mathrm{vehicle}\;\mathrm{types}$$

The likelihood function becomes:

(3) $$L\left(\beta \right)=\underset{n\in N}{\Pi }\underset{t\in T}{\Pi }\underset{v\in K}{\Pi }{\left(P_{\mathrm{vnt}}\right)}^{y_{\mathrm{vnt}}}$$

where $y_{\mathrm{vnt}}$ = 1, if firm n chose vehicle type v for shipment *t* and zero otherwise.

### Mixed Logit Model

The mixed logit model with random coefficients is used with a MNL structure. As multiple shipments are made by same firm, inter-firm draws are used to introduce correlation among the choices made by the same firm. The utility equation remains the same as for the MNL. The likelihood function for firm n choosing vehicle type v becomes the product of logit formulas for each shipment *t* (*40*):

(4) $$L_{\mathrm{vn}}\left(\beta \right)=\underset{t\in T}{\Pi }\left[\frac{e^{V_{\mathrm{vnt}}}}{\sum_{k\in K}e^{V_{\mathrm{knt}}}}\right]$$

The choice probability of vehicle type *v* for firm n becomes (*40*):

(5) $$P_{\mathrm{vn}}=\int L_{\mathrm{vn}}\left(\beta \right)f(\beta )d\beta$$

f(β) is the density of parameters β . Normal distribution is used as a mixing distribution. The equation for the random coefficient becomes:

(6) $$\beta =\bar{\beta }+\sigma *\eta$$

where $\bar{\beta }$ is the mean and σ is the standard deviation of the random coefficient which are estimated. η is standard normal and 100 inter-firm Halton draws are used.

## Results

### Random Forest

**Figure 5.**
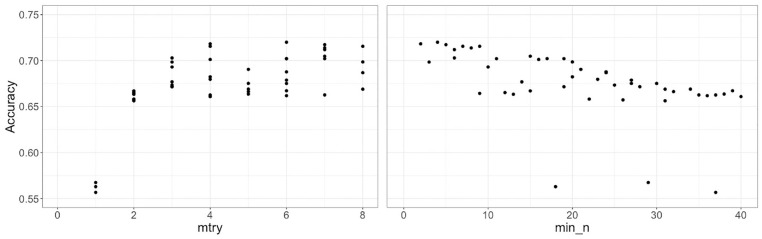
Random forest hyperparameter tuning results for initial 50 randomly selected values.

The hyperparameters *mtry* and *min_n* are tuned using a grid-based approach (*41*) in two steps. First, a grid size of 50 representing 50 pairs of randomly selected values of the two hyperparameters are tested. The variables commodity and industry type with at least ten observations for each vehicle type are used in the model. The hyperparameter *mtry* has a range from [1, q], where q is the number of independent variables. For, hyperparameter *min_n* we tested approximately all values from 2 to 40, as we observe a decreasing trend (Figure 5). Second, based on the hyperparameter results of the first step, the grid is re-defined within the range of best-performing hyperparameter values to select the best combination of the hyperparameter values. Figure 5 shows the model performance results of the initial 50 randomly selected values of *mtry* and *min_n*, respectively. Every combination from the selected range of the two hyperparameters is tested in the second step.

**Figure 6.**
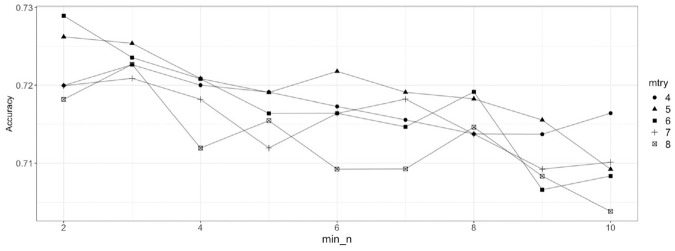
Random forest hyperparameter tuning results for the refined grid.

In Figure 5, each value of *mtry* generates multiple values of accuracy, because each value of *mtry* is tested with different values of *min_n*. From Figure 5, values of *mtry* ranging from 4 to 8 are selected for the second step because of relatively better performance than lower values. Figure 5 shows that the model performance is high at low values of *min_n*. Therefore, values of *min_n* ranging from 2 to 10 are selected for the second step. The refined grid consists of 45 pairs of the two hyperparameters. Figure 6 shows the results of model performance for the refined grid. From Figure 6, the values of hyperparameter *mtry* and *min_n* are selected to be 6 and 2 for the final model, respectively. Intuitively, it means that out of all explanatory variables in the model, six variables are randomly selected as the candidate variables for the node split and the minimum size of the node is 2, which corresponds to a larger tree size. The accuracy with the selected hyperparameters is approximately 72.9%.

#### Variable Importance

**Figure 7.**
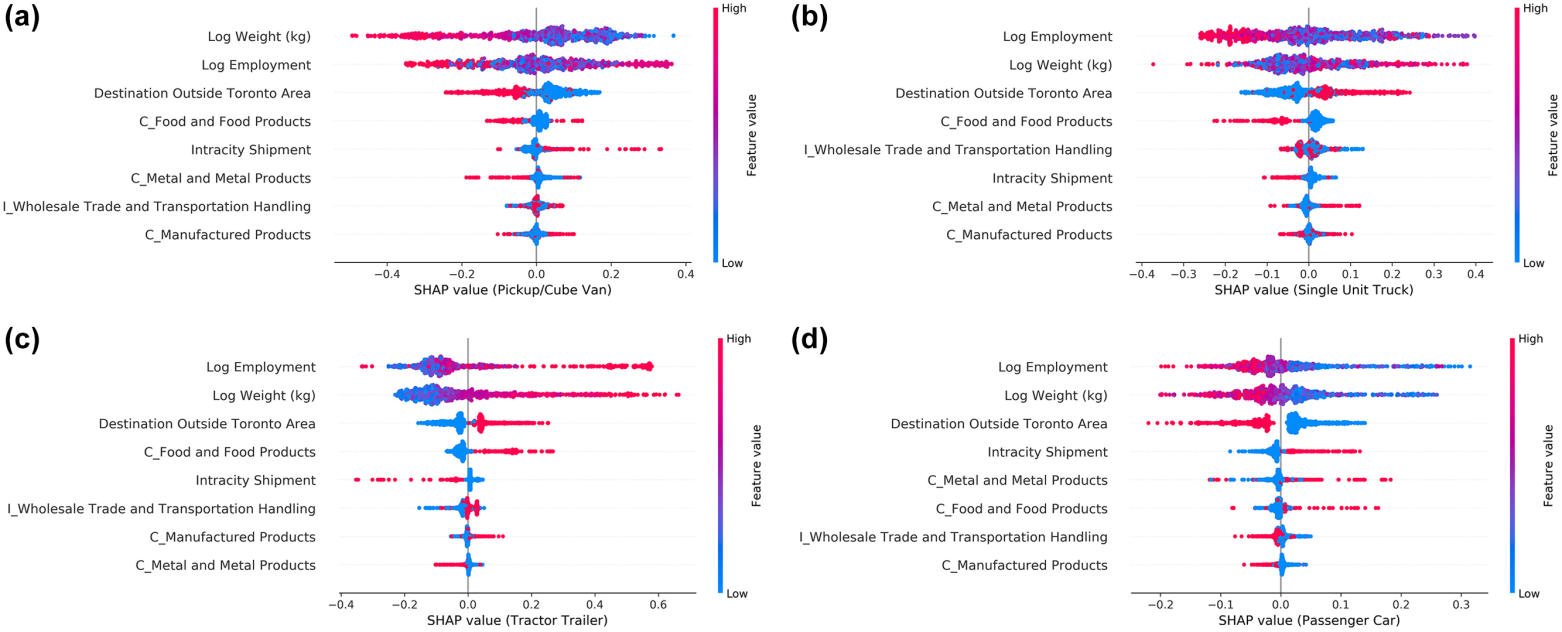
Variable importance for each vehicle type: (a) variable importance for pickup/cube van, (b) variable importance for single unit truck, (c) variable importance for tractor trailer, and (d) variable importance for passenger car.

Figure 7 shows SHAP summary plots for each vehicle type. The plots show the global impact of each independent variable on vehicle types. The independent variables are sorted by the order of importance. The letter “I” in the variable names represents industry type and “C” represents commodity type. Figure 7 shows that weight and employment are the two most important variables affecting the choice of vehicle type. Figure 7 shows that increasing weight (red values) is associated with lower chance of selecting pickup/cube van and passenger car and higher chance of selecting tractor trailer. A similar effect is observed for employment. For single-unit truck, Figure 7*b* shows a reduced chance of selection by small-size companies and an increased chance with higher weight. However, the higher weight is observed throughout the spectrum. For intracity shipments, Figure 7 shows that pickup/cube van has higher chance to be selected, whereas single-unit truck and tractor trailer have lower chance. Shipments with destination outside of the Toronto Area have an opposite effect compared with intracity shipments. Figure 7 shows that food and food products have higher chance to be transported via tractor trailer or passenger car and a lower chance via single-unit truck. This could be because of weight distribution of food and food products which could supply a large retail/wholesale store or a small shop. However, a clear pattern is not observed for pickup/cube van. Manufactured products have higher chance of transportation via tractor trailer and a lower chance with passenger car. The pattern is unclear for pickup/cube van and single-unit truck. Wholesale trade and transportation handling industries have higher chance of transporting shipments using tractor trailer and a lower chance using passenger car. The pattern is unclear for pickup/cube van and single-unit truck, possibly because of a non-linear relationship. Moreover, the general trend of longer tails on one side for some variables shows that extreme values of the variables do not have the same effect, also indicating non-linear relationships. For example, in the case of tractor trailer, a longer tail to the right side but not to the left side for weight shows that extreme values of weight can significantly increase the chance of tractor trailer selection but cannot significantly reduce the chance of selection.

#### Model Predictions

Table 3 shows the RF model prediction results on the validation data. Category accuracy is calculated by dividing number of correct predictions by total number of observed vehicle types. Table 3 shows that pickup or cube van is over predicted by 42% with accuracy of predictions around 65%. Similarly, single-unit truck is under-predicted by around 20% with a prediction accuracy of approximately 32%. Passenger car is under-predicted by around 44% and accuracy of predictions is around 31%. The model prediction performance for vehicle type tractor trailer is the best among other vehicle types, with accuracy of around 67%. The overall model accuracy is approximately 50%.

**Table 3.** Random Forest Model Prediction Results on the Validation Data

Vehicle type	Observed	Predicted	Over/under prediction (%)	Number of correct predictions	Category accuracy (%)
Pickup or cube van	102	145	42.2	66	64.7
Single-unit truck	106	85	−19.8	34	32.1
Tractor trailer	69	68	−1.4	46	66.7
Passenger car	48	27	−43.8	15	31.2
Total	325	325	na	161	49.5

Note: na = not applicable

### Multinomial Logit Model

The MNL model is estimated while keeping the alternative pickup or cube van as a reference alternative with utility of zero. Table 4 shows the model estimation results. The employment coefficients show that, compared with other vehicle types, large firms are more likely to use a tractor trailer than a passenger car.

**Table 4.** Multinomial Logit Model Estimation Results

Variable description	Estimate	T-ratio
Constant: Single-unit truck (SUT)	−2.91	−11.59
Constant: Tractor and trailer (TT)	−8.32	−14.62
Constant: Passenger car (PC)	−0.54	−1.74
Log employment: TT	0.72	7.44
Log employment: PC	−0.50	−4.70
Log weight: SUT	0.41	9.75
Log weight: TT	0.88	14.41
Log weight: PC	−0.14	−2.37
Intracity shipment: TT	−0.83	−2.51
Intracity shipment: PC	1.23	5.06
Destination outside Toronto area: SUT	1.64	8.63
Destination outside Toronto area: TT	2.44	9.05
Commodity: Manufactured products: SUT	0.69	3.37
Commodity: Metal and metal products: SUT	0.98	4.48
Commodity: Wood, pulp, and paper: SUT	1.14	3.57
Commodity: Textiles: SUT	3.15	3.82
Commodity: Road vehicles and parts: SUT	0.95	3.12
Commodity: Food and food products: TT	2.51	8.02
Commodity: Manufactured products: TT	0.53	1.98
Commodity: Metal and metal products: PC	0.72	2.54
Industry: Retail trade: SUT	0.76	3.28
Industry: Wholesale trade and transportation handling: TT	0.48	2.08
Model performance		
L(0)	−1544.33	
L(β)	−950.58	
Adjusted Rho-square	0.37	
BIC	2055.50	

*Note*: BIC = Bayesian information criterion.

Compared with other industry types, wholesale trade and transport handling industries are more likely to use a tractor trailer whereas firms belonging to retail trade are more likely to use a single-unit truck. Table 4 also show that firms are more likely to transport shipments with large weight using a single-unit truck or a tractor trailer as compared with a passenger car. As compared with other commodity types, food products are more likely to be transported using tractor trailer, whereas wood, pulp, and paper are more likely to be transported using single-unit truck. Manufactured products are more likely to be transported using single-unit truck or tractor trailer. Metal and metal products, textiles, and road vehicles and parts are also likely to be transported using single-unit truck as compared with other commodity types. Intracity shipments are more likely to be transported using passenger car and less likely using tractor trailer, whereas shipments destined outside of Toronto Area are more likely to be transported using larger vehicle, that is, single-unit truck and tractor trailer.

The estimated MNL model is applied to the validation data. Table 5 shows the model prediction results on the validation data. The category accuracy column shows that tractor trailer is most accurately predicted, followed by pickup/cube van, single-unit truck, and passenger car. The overall model prediction accuracy is 41.7%.

**Table 5.** Multinomial Logit Model Prediction Results on the Validation Data

Vehicle type	Observed	Predicted	Over/under prediction (%)	Number of correct predictions	Category accuracy (%)
Pickup or cube van	102	123	21.0	52	50.6
Single-unit truck	106	99	−6.3	37	34.8
Tractor trailer	69	73	5.4	38	55.1
Passenger car	48	30	−38.4	9	18.8
Total	325	325	na	136	41.7

Note: na = not applicable

### Mixed Logit Model

The mixed logit model is also estimated while keeping the alternative pickup or cube van as a reference alternative with utility of zero. The coefficient of weight for tractor trailer and passenger car is used as a random coefficient. Table 6 shows the model estimation results. All the variables have similar signs, but increased magnitudes compared with the MNL model. Moreover, some variables become insignificant and therefore are removed from the model. The interpretation of the coefficients is similar to the MNL model. The mean and standard deviation of weight for tractor trailer and passenger car are statistically significant, indicating that the parameters vary between firms. The sensitivity of weight for selecting passenger car or tractor trailer varies among firms. The overall model performance has improved, as compared with the MNL model, with an increase of rho-squared and reduction in BIC value.

**Table 6.** Mixed Logit Model Estimation Results

Variable description	Estimate	T-ratio
Constant: Single-unit truck (SUT)	−3.25	−11.42
Constant: Tractor and trailer (TT)	−15.08	−6.93
Constant: Passenger car (PC)	−0.47	−1.20
Log employment: TT	1.81	4.40
Log employment: PC	−0.51	−3.69
Log weight: SUT	0.45	10.02
Intracity shipment: TT	−2.01	−3.12
Intracity shipment: PC	1.41	4.55
Destination outside Toronto area: SUT	1.79	8.88
Destination outside Toronto area: TT	3.53	6.13
Commodity: Manufactured products: SUT	0.79	3.68
Commodity: Metal and metal products: SUT	1.12	4.62
Commodity: Wood, pulp, and paper: SUT	1.54	4.21
Commodity: Textiles: SUT	3.44	3.97
Commodity: Road vehicles and parts: SUT	0.97	3.01
Commodity: Food and food products: TT	3.77	3.99
Mean log weight: TT	1.21	7.61
Standard deviation log weight: TT	0.81	5.67
Mean log weight: PC	−0.44	−3.79
Standard deviation log weight: PC	0.61	5.82
Industry: Retail trade: SUT	0.97	3.76
Industry: Wholesale trade and transportation handling: TT	1.33	1.73
Model performance		
L(0)	−1544.33	
L(β)	−831.53	
Adjusted Rho-square	0.45	
BIC	1817.41	

*Note*: BIC = Bayesian information criterion.

The estimated mixed logit model is applied to the validation data. Table 7 shows the model prediction results on the validation data. The category accuracy column shows that pickup/cube van is most accurately predicted, followed by tractor trailer, single-unit truck, and passenger car. The overall model prediction accuracy is 39.9%.

**Table 7.** Mixed Logit Model Prediction Results on the Validation Data

Vehicle type	Observed	Predicted	Over/under prediction (%)	Number of correct predictions	Category accuracy (%)
Pickup or cube van	102	121	18.9	51	50.3
Single-unit truck	106	100	−5.2	36	34.1
Tractor trailer	69	67	−3.3	33	47.2
Passenger car	48	37	−23.9	10	20.0
Total	325	325	na	130	39.9

Note: na = not applicable

## Discussion and Conclusion

This study develops machine learning and discrete choice models to study the choice of vehicle type by firms in the Toronto Area. This study explicitly models the choice between vehicle types for road transport shipments. The development of a RF model shows that the model performance varies with different values of hyperparameters and therefore warrants hyperparameter tuning. For comparison, a MNL model is developed for the vehicle type choice. As the data represent a panel structure, a mixed MNL model is also developed to represent the correlation of choices made by the same firm. Interestingly, the SHAP-based variable importance and coefficients of MNL and mixed MNL show similar results. For example, Figure 7*c* shows that higher weight corresponds to high chance of tractor trailer to be selected, and Tables 4 and 6 show positive coefficient of weight for tractor trailer. Similarly, for intracity shipments, passenger car has positive coefficient for both MNL and mixed MNL models, and SHAP summary plot (Figure 7*d*) also shows positive SHAP values for intracity shipments. This shows that SHAP summary plots present reasonable explanation of independent variables as compared with other methods for calculating variable importance. Among discrete choice models, mixed logit model has better model performance based on rho-squared and BIC values. The three models are applied on the validation data and compared based on their prediction accuracy. Tables 3, 5, and 7 show the model application results on the validation data by RF, MNL, and mixed MNL model, respectively. The comparison shows that, in relation to prediction accuracy, the RF model outperforms both MNL and mixed MNL model, with an overall prediction accuracy of 49.5%. This finding is in line with the RF application in mode choice modeling in passenger transportation. The prediction accuracy of MNL and mixed MNL model is 41.7% and 39.9%, respectively. The mixed MNL model prediction accuracy is slightly lower than MNL despite an increase of adjusted rho-square value from 0.37 to 0.45. This could be explained by the lower number of explanatory variables in the mixed MNL model than the MNL model. Some variables used in the MNL model become statistically insignificant and are therefore removed from the mixed MNL model. The comparison of vehicle type predictions shows interesting results. For the three models, tractor trailer and pickup/cube van are the two most accurately predicted, followed by single-unit truck and passenger car. The RF model outperforms the MNL and mixed MNL models in relation to prediction accuracy, therefore, it can be concluded that RF model is recommended for further development and potential application.

The model prediction performance of all of the models estimated in this study can be improved by increasing the sample size and including additional alternative specific variables that affect vehicle type choice. Future data collection efforts in the Toronto Area should consider collecting information about the cost and time of shipment transportation and attributes of the receiver firm. Another important avenue of future research is in the SHAP values for variable importance. SHAP-based variable importance provides importance of variables as well as their positive or negative impacts. However, future research could study the use of SHAP values in comparison with coefficients of discrete choice models. This could overcome the challenges posed by the RF method for policy analysis compared with traditional discrete choice models. Moreover, future studies could also compare the results of multiple machine learning classifiers with RF and discrete choice methods.
